# Microbial network properties and functional gene diversity drive soil multifunctionality during biocrust succession

**DOI:** 10.3389/fmicb.2025.1656706

**Published:** 2025-09-04

**Authors:** Yawen Jiang, Xuexia Zhang, Mingjie Li, Jing Yang, Yuqing Zhang, Shugao Qin, Wei Feng

**Affiliations:** ^1^Yanchi Research Station, School of Soil and Water Conservation, Beijing Forestry University, Beijing, China; ^2^School of Soil and Water Conservation, Beijing Forestry University, Beijing, China; ^3^Key Laboratory of State Forestry Administration on Soil and Water Conservation, Beijing Forestry University, Beijing, China; ^4^State Key Laboratory of Efficient Production of Forest Resource, Beijing Forestry University, Beijing, China; ^5^Engineering Research Center of Forestry Ecological Engineering, Ministry of Education, Beijing Forestry University, Beijing, China

**Keywords:** biocrusts, soil multifunctionality, microbial co-occurrence networks, microbial taxonomic diversity, microbial functional gene diversity

## Abstract

**Introduction:**

Biocrusts are critical components of desert ecosystems, performing vital functions including soil stabilization, nutrient enrichment, and regulation of carbon (C) and nitrogen (N) cycles. This study investigated the microbial mechanisms underlying biocrust-mediated soil multifunctionality (SMF) in the Mu Us Desert by comparing algal-, lichen-, and moss-dominated crusts.

**Methods:**

We systematically sampled biocrust layers and underlying subcrust soils (0–5 cm depth), employing metagenomic sequencing and co-occurrence network analysis to characterize microbial community structures and functional properties. SMF was quantified using an integrative index based on ten parameters associated with C, N, and phosphorus (P) cycling processes.

**Results:**

Results revealed significant variation in SMF among biocrust types, with moss crusts exhibiting the highest level, followed by lichen and algal crusts. Microbial community characteristics indicated that although lichen crusts showed the highest taxonomic diversity and network complexity, moss crusts showed a significant positive correlation with SMF. Network topological parameters, particularly network density within the biocrust layers, correlated significantly positively with SMF (p < 0.05), contrasting with the non-significant relationship observed for taxonomic diversity. Functional gene analysis revealed that the diversity of C degradation and N cycling genes exhibited a significant positive correlation with SMF.

**Discussion:**

Our findings demonstrate that biocrusts enhance SMF primarily by mediating both direct and indirect effects on N cycling functional gene diversity and microbial network complexity. This study underscores the critical role of functional gene diversity in driving biocrust-mediated ecological functions in desert ecosystems and provides a theoretical framework for developing sustainable land management and ecological restoration strategies in drylands.

## 1 Introduction

Biocrusts represent vital components of desert ecosystems, dominating vegetation-free surfaces and occupying > 40% of ground cover in these regions, equivalent to approximately 12% of the Earth’s continental land area (Rodriguez-Caballero et al., 2018; Li Y. H. et al., 2021). Biocrusts comprise photoautotrophic communities (cyanobacteria, algae, lichens and bryophytes) that co-occur with heterotrophic bacteria, archaea and fungi, forming cohesive surface layers on arid soils (Rodriguez-Caballero et al., 2018). Based on biological composition, biocrusts are categorized into algal-, lichen-, and moss-dominated types, each exhibiting unique ecological functions due to divergent physicochemical properties (Li X. R. et al., 2021; Li Y. H. et al., 2021). Crucially, biocrusts enhance soil physical structure, promote nutrient accumulation, and elevate carbon (C) and nitrogen (N) stocks in desert ecosystems. Through these synergistic effects, they enhance soil multifunctionality (SMF) (Billings et al., 2003; Housman et al., 2006; Bu et al., 2013). Biocrusts do not function as isolated ecosystem units; their formation, stability, and functionality are tightly coupled with and interact dynamically with the underlying subcrust soil (Dou et al., 2022; Liu Q. et al., 2024). Biocrust development critically depends on physical support, water storage, and nutrient supply provided by the subcrust soils. Simultaneously, biocrusts profoundly modify subcrust soils physicochemical and biological properties through microclimate alteration, enhanced organic matter input, and regulation of water infiltration and nutrient cycling (Eldridge et al., 2020; Mackelprang et al., 2022; Li et al., 2023).

SMF defined as the capability of soils to simultaneously sustain multiple ecological processes such as nutrient cycling, organic carbon transformation, and fertility maintenance – is fundamental to ecosystem stability (Deng et al., 2024; Hu X. G. et al., 2024). Although considerable advances have elucidated SMF in forest, agricultural, and grassland ecosystems, understanding of biocrusts – mediated SMF in deserts remains limited. Current knowledge faces two principal constraints: First, while microorganisms are primary drivers of biocrust development and function, the specific mechanisms through which microbial communities regulate SMF remain unresolved (Zhai et al., 2024). Second, existing studies predominantly focus on microbial taxonomic diversity, neglecting the governing roles of microbial interaction networks and functional gene composition in determining SMF (Xiao et al., 2023). These critical gaps hinder efforts to enhance the functional stability of desert ecosystems.

As core components of biocrusts, microorganisms directly regulate soil biogeochemical processes through their diversity and functional traits (Maier et al., 2018). Enhanced microbial diversity typically increases SMF via functional redundancy and complementarity (Wagg et al., 2019). However, studies in agricultural ecosystems demonstrate that microbial interactions are stronger predictors of SMF than taxonomic diversity alone (Jiao et al., 2022). Furthermore, functional genes (e.g., those involved in C, N, and P cycling) drive the synergistic enhancement of SMF by underpinning multiple individual processes, such as nutrient cycling and organic matter decomposition (Liu L. et al., 2024; Liu Q. et al., 2024). Divergent effects on SMF are observed across biocrust types: algal crusts primarily facilitate carbon and nitrogen accumulation, whereas moss crusts enhance phosphorus mineralization; collectively, these contribute to sustaining overall SMF (Mager and Thomas, 2011; Xu et al., 2023). Nevertheless, the drivers of SMF exhibit substantial variation across different ecosystems, and a generalizable framework for its comprehensive assessment remains elusive (Byrnes et al., 2013). Notably, within desert ecosystems, the mechanisms by which biocrusts influence SMF are poorly understood. In particular, the relative contributions of taxonomic diversity, microbial network interactions, and functional gene assemblages require quantitative assessment.

This investigation focuses on algal-, lichen-, and moss-dominated biocrusts in the Mu Us Desert. Utilizing a stratified sampling design (biocrust layer and the underlying subcrust layers) combined with metagenomic sequencing, we examine microbial community structure and functional gene profiles across biocrust types. Specifically, the study aims to address the following: (1) Assess differences in SMF among biocrust types and between biocrusts layer and their underlying soils; (2) Determine how microbial taxonomic diversity and co-occurrence network topology within biocrusts relate to SMF; (3) Evaluate the influence of functional gene composition and diversity in biocrusts on SMF. By integrating these perspectives, this research advances our understanding of the mechanisms by which biocrusts modulate ecosystem functioning in the Mu Us Desert. The findings not only address critical knowledge gaps regarding microbial drivers of SMF but also provide a scientific basis for managing and accessing soil ecosystem health in dryland regions.

## 2 Materials and methods

### 2.1 Study site

This research was conducted at the Yanchi Research Station (37 °40′–38 °10′N, 106 °30′–107 °41′E), located on the southwestern fringe of the Mu Us Desert in northern China at 1,530 m elevation (Zhu et al., 2024). The site experiences a semi-arid mid-temperate continental monsoon climate. Meteorological records (1954–2022) from Yanchi Meteorological Station (~20 km northeast) indicate a mean annual temperature of 8.4 °C and precipitation of 292 mm, with ~80% occurring during the growing season (May–September). Annual potential evaporation averages 2024 mm. Soils are sandy-textured, with bulk density of 1.54 ± 0.08 g cm^−3^ and field capacity of 20.31 ± 3.33% (0–20 cm depth) (Jia et al., 2018; Hao et al., 2023). Dominant vegetation includes *Artemisia ordosica*, *Hedysarum mongolicum*, *Salix psammophila*, and *Caragana korshinskii*, with biocrusts colonizing plant interspaces across xeric-to-mesic microhabitats. Biocrust communities comprise algae (*Oscillatoria chlorina*, *Microcoleus vaginatus*), lichens (*Collema tenax*), and mosses (*Bryum argenteum*) (She et al., 2024).

### 2.2 Experiment design and sampling

Three biocrust types (algal, lichen, moss) were sampled at Yanchi Research Station in June 2017. Within three spatially segregated 1-km^2^ plots (one per biocrust type), we established three 50 × 50 m subplots (*n* = 3 per biocrust type; minimum 50 m spacing). Biocrust samples (1–2 cm natural thickness) and underlying subcrust soils (5 cm below crust interface) were collected using a diagonal intersection strategy (5 points: center + corners). At each subplot, 12 biocrust layer samples were randomly obtained using sterilized cutting rings (inner diameter: 9 cm; depth: 1 cm), which were homogenized into one composite sample per subplot (yielding 9 composite biocrust samples). Subcrust soils were similarly collected and composited using the same method, yielding 9 composite soil samples. This resulted in a total of 18 composite soil samples. Plant litter was removed from biocrust surfaces prior to sampling, and all equipment was sterilized with 75% ethanol between samples. Samples were immediately stored in cooling boxes (4 °C) and transported to the laboratory within 4 h. Each composite sample was then divided into three aliquots: (1) fresh subsamples for determining physicochemical properties and enzyme activities; (2) Flash-frozen (−80 °C) for DNA sequencing; (3) Refrigerated (4 °C) for microbial biomass.

### 2.3 Soil physicochemical properties determination

The fresh subsamples were used for physicochemical properties. Soil organic carbon (SOC) was quantified by K₂SO₄ extraction. Total nitrogen (TN) and phosphorus (TP) were determined using Kjeldahl micro-analysis (FOSS 2200) and molybdenum-blue spectrophotometry, respectively. Inorganic nitrogen fractions were measured as: NO₃^−^-N by UV spectrophotometry and NH₄^+^-N by continuous flow analysis after CaCl₂ extraction. Enzyme activities were assessed calorimetrically: invertase via 3,5-DNS method, urease by phenol-hypochlorite, and alkaline phosphatase using phenyl phosphate disodium substrate. The soil samples stored at 4 °C used to determine microbial biomass carbon (MBC) and nitrogen (MBN) via chloroform fumigation-K₂SO₄ extraction.

### 2.4 Shotgun metagenome sequencing

Soil samples stored at −20 °C were subjected to metagenomic sequencing on an Illumina HiSeq platform (Novogene Co., Beijing). Raw sequences underwent adapter trimming and quality filtering (discarding reads <40 bp, average quality score ≤38, or exceeding thresholds for N-base content/adapter overlap). High-quality reads were assembled into contigs using Megahit v1.2.9, retaining contigs ≥500 bp for downstream analysis. Open reading frames (ORFs) were predicted from contigs using Metagene Mark v3.38. For taxonomic annotation, ORFs were aligned against the NCBI NR database using BLASTP (e-value ≤ 1e^−5^). The lowest common ancestor (LCA) algorithm assigned classifications at kingdom to species levels, with gene abundance calculated per taxon. Functional annotation was performed via DIAMOND v2.0.15 against KEGG (2023 release) using best-hit criteria (e-value ≤ 1e^−5^). KEGG Orthology (KO), pathway, and module abundances were quantified based on aligned gene counts following Liu et al. (2023).

### 2.5 Soil multifunctionality calculations

For this study, ten key indicators of ecosystem functioning were selected to characterize soil multifunctionality. These indicators include SOC, TN, TP, MBC, MBN, NO₃^−^-N, NH₄^+^-N, urease activity, alkaline phosphatase activity, and invertase activity. Closely linked to the storage and cycling of C, N, and P, these metrics collectively provide a robust representation of multiple ecosystem functions, including nutrient sequestration and utilization, soil fertility, and biogeochemical cycles (Hu W. et al., 2024; Hu X. G. et al., 2024). Each of these ten indicators was standardized using the Z-score method (Equation 1), followed by the calculation of soil multifunctionality. The formula for Z-score standardization is as follows:

(1) $$Z-\text{score}=\frac{F-\text{MeanF}}{\mathrm{SDF}}$$

where: *F* is observed value of the indicator, *MeanF* is mean value of the indicator across samples, *SDF* is standard deviation of the indicator across samples. The soil multifunctionality index of each plot was obtained by averaging the Z-scores of the ten different parameters (Sun et al., 2021). The calculation formula is as follows (Equation 2):

(2) $$\mathrm{SMF}=\frac{1}{10}\sum_{i=1}^{10}Z-\text{scores}$$

### 2.6 Soil microbial community and functional characteristics

Microbial taxonomic α-diversity (Shannon, species richness, Chao1) and functional gene α-diversity (Shannon, Chao1, Simpson for C/N/P cycling genes) were calculated using *vegan* v2.6-4 package in R v.4.4.1. Co-occurrence networks were constructed from species-level abundance data (relative abundance > 0.002%; occurrence frequency >1/6) via the *WGCNA* v1.72 package in R v.4.4.1 (Langfelder and Horvath, 2008). Spearman’s rank correlation coefficients (*r*) were computed using the *psych* v2.5.6 package in R v.4.4.1 Nodes and edges were retained only when meeting the threshold criteria of |*r*| > 0.55 with statistical significance of *p* < 0.01 (FDR-corrected) (Benjamini and Hochberg, 1995). Weighted undirected networks were constructed from the correlation matrices using the *igraph* v.2.2.4 package in R v.4.4.1. Edge weights were assigned based on correlation coefficients, and categorical attributes were added to nodes. Common topological metrics were subsequently calculated. Network visualization was implemented in Gephi 0.9.2. For each sampling site, sub-networks were extracted from the integrated network using the *subgraph* function in *igraph* v.2.2.4, from which node counts, edge counts, and network density were derived (Zhang B. et al., 2018; Wagg et al., 2019; Qin et al., 2021).

### 2.7 Statistical analysis

Differences in soil parameters and microbial diversity across biocrust types/layers were assessed using ANOVA with LSD post-hoc tests (α = 0.05). Parametric assumptions (normality: Shapiro–Wilk; homogeneity: Levene’s test) were verified, with log-transformation applied to non-normal variables. Pearson correlations quantified relationships between microbial characteristics and SMF. To clarify the contributions of microbial communities versus functional traits to ecosystem multifunctionality, we conducted variance partitioning analysis (VPA) using the *varpart* function in the *vegan* v2.6-4 package in R v.4.4.1, followed by rigorous validation of unique components through partial redundancy analysis (pRDA) combined with 999 permutations. Structural equation modeling (SEM) elucidated direct/indirect pathways linking microbial parameters to ecosystem multifunctionality. The SEM was deemed acceptable if it satisfied all the following goodness-of-fit criteria: 0 ≤ *Chisq*/*df* ≤ 2 with 0.05 < *p* ≤ 1.00; 0.90 ≤ Goodness of Fit Index (GFI) ≤ 1.00; 0.90 ≤ Comparative Fit Index (CFI) ≤ 1.00; and 0.90 ≤ Normed Fit Index (NFI) ≤ 1.00.

## 3 Results

### 3.1 Differences in soil properties and multifunctionality of biocrusts

Among all measured parameters, NO₃^−^-N and urease concentrations were lower in the moss crust layer compared to its subcrust layer (Supplementary Figure S1). In contrast, all other indicators were significantly higher in the crust layers than in the subcrust layers. Within the biocrust layers, SOC, TN, and MBC contents differed significantly among the three biocrust types (*p* < 0.01, Supplementary Figure S1). However, the subcrust layers, exhibited no significant differences in TN, TP, MBC, MBN, NH₄^+^-N, urease, or invertase across the biocrust types.

**Figure 1:**
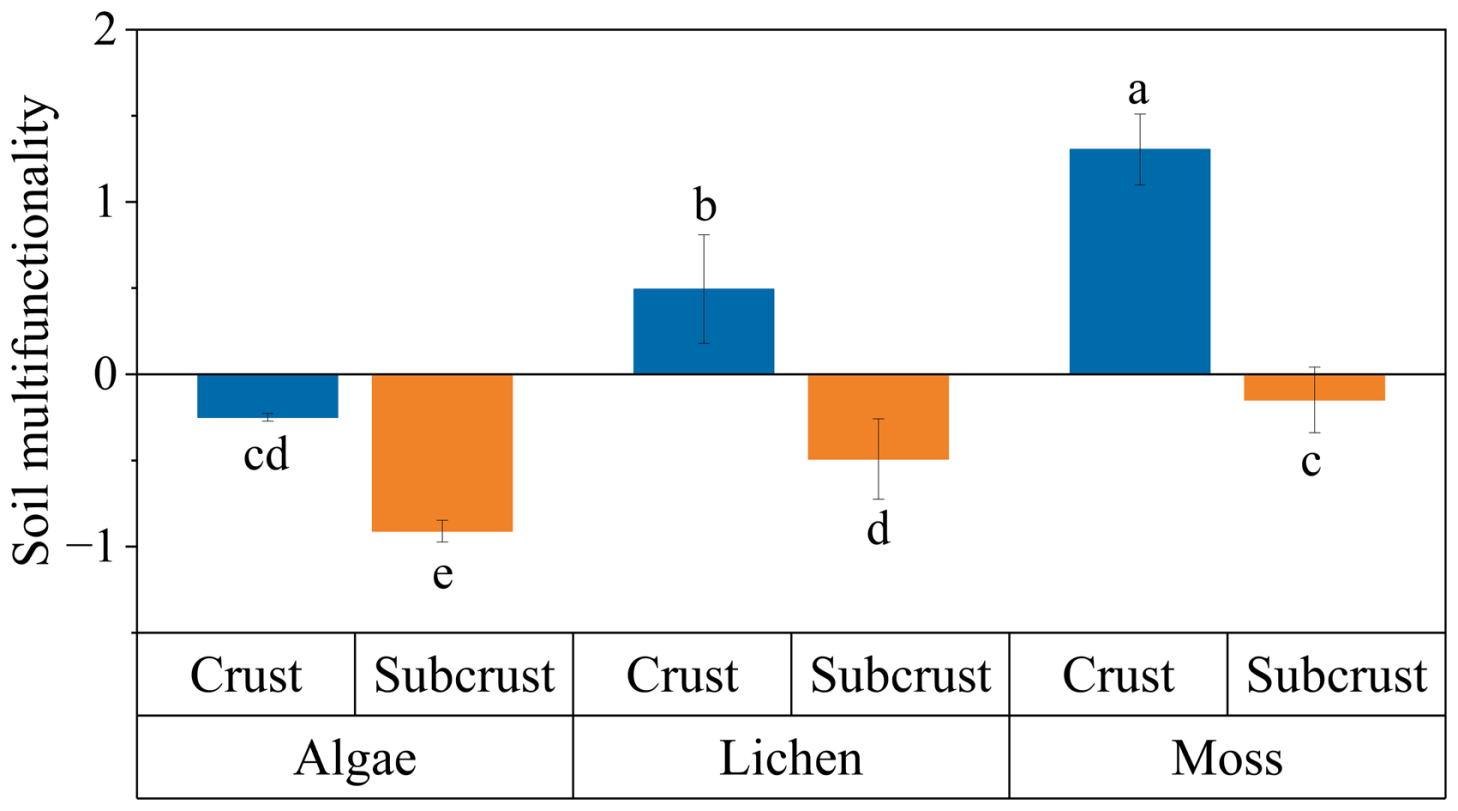
Soil multifunctionality of different biocrusts. Different lowercase letters indicate significant differences (*p* < 0.05), data are shown as mean ± SE.

SMF was significantly higher in biocrust layers than in subcrust layers (*p* < 0.05) and differed significantly among biocrust types (*p* < 0.05, Figure 1). Moss crusts had the highest SMF, followed by lichen and algal crusts, in both biocrust and subcrust layers. Positive SMF values (>0) were only observed in the lichen and moss crusts layers. Quantitative values for biocrust layers SMF were −0.25 ± 0.02 (algae), 0.49 ± 0.32 (lichen), and 1.31 ± 0.21 (moss). Corresponding values for subcrust layers were: −0.91 ± 0.06, −0.49 ± 0.23, and −0.15 ± 0.19.

### 3.2 Difference in microbial taxonomic and functional properties

**Figure 2:**
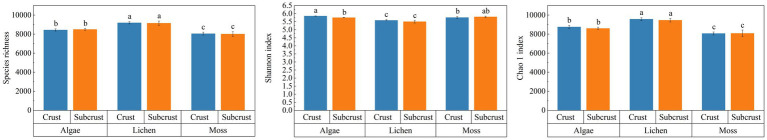
Soil microbial taxonomic diversity in different biocrusts. Different lowercase letters indicate significant differences (*p* < 0.05), data are shown as mean ± SE.

Species richness and the Chao1 index were significantly higher in both lichen crust layers and their subcrust layers compared to the corresponding layers in algal crusts and moss crusts (Figure 2). Conversely, Shannon diversity indices showed an inverse pattern: algal crust layers had the highest values, followed by moss crust layers, while lichen crust layers had significantly lower values. Across the three biocrust types, there were generally no significant differences in these taxonomic diversity indices between biocrust layers and subcrust layers. However, an exception was observed in algal crusts, where the biocrust layer had a significantly higher Shannon index than its subcrust layer.

**Figure 3:**
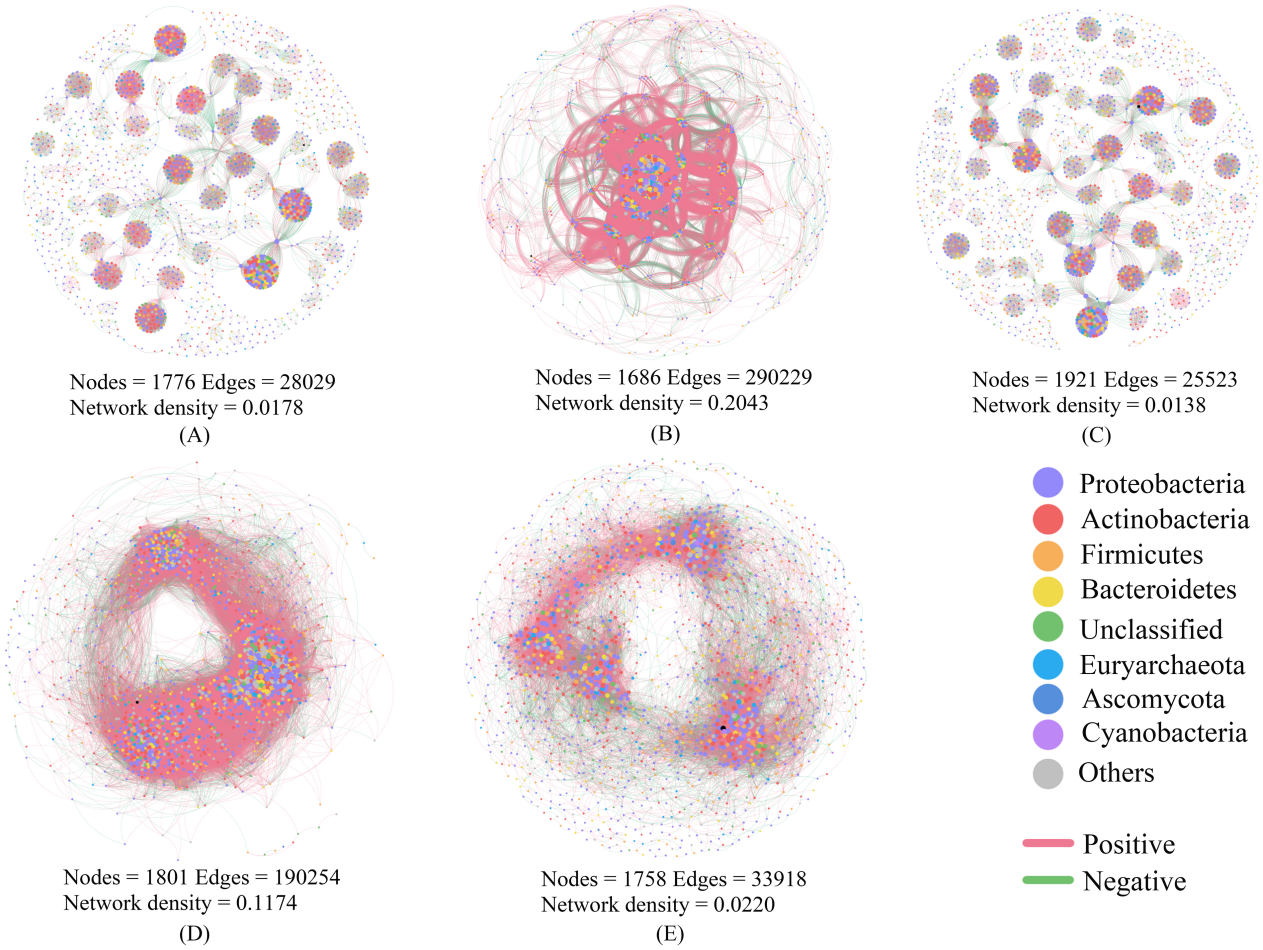
Microbial co-occurrence networks of soil microorganisms with different biocrusts. **(A)** algal crusts, **(B)** lichen crusts, **(C)** moss crusts, **(D)** biocrust layer, **(E)**: subcrust layer.

Co-occurrence network complexity, as indicated by network density, was significantly higher in lichen crusts (0.2043) than in algal crusts (0.0178) or moss crusts (0.0138) (Figure 3). Furthermore, the biocrust layer (0.1174) exhibited greater network complexity than its respective subcrust layer (0.0220). Across all networks, positive interactions between nodes consistently exceeded 50%, indicating predominantly cooperative relationships among soil microbial species. The phyla Proteobacteria, Actinobacteria, and Firmicutes (each with relative abundance >10%) were key components contributing to network stability.

Comparative analysis revealed significant differences (*p* < 0.05) between biocrust layers and subcrust layers in the Shannon and Simpson indices of microbial functional genes associated with C fixation, C degradation, and P cycling (Supplementary Figure S2). Notably, the subcrust layer exhibited greater diversity in C fixation genes than the biocrust layer. Among the three biocrust types, significant differences in the Shannon and Simpson indices of functional genes were only observed for N cycling genes (*p* < 0.05).

### 3.3 Influence of microbial taxonomic and functional properties on soil multifunctionality

**Figure 4:**
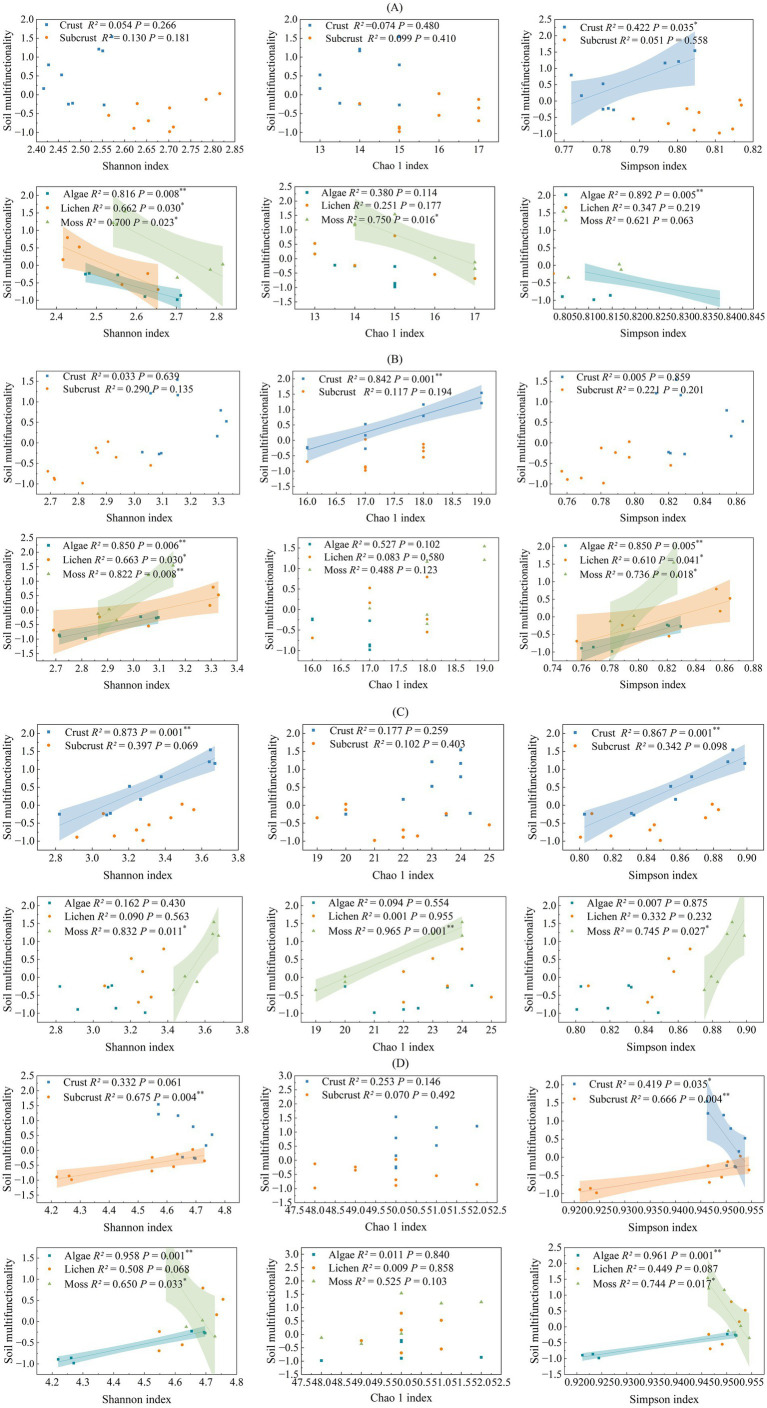
Relationship between soil multifunctionality (Z-score) and soil microbial functional diversity. Shadow regions indicate 95% confidence intervals around the regressions. *R*^2^, Coefficient of determination; * *p* < 0.05; ** *p* < 0.01. **(A)** carbon fixation, **(B)** carbon degradation. **(C)** nitrogen cycle, **(D)** phosphorus cycle.

Pearson correlation analysis revealed no significant correlation between SMF and taxonomic α-diversity of biocrusts (Supplementary Figure S3). However, in biocrust layers, network density showed a significant positive correlation with SMF (*p* < 0.05), contrasting with a significant negative correlation (*p* < 0.05) in subcrust soils. In biocrust layers, α-diversity of C fixation gene (Simpson index), C degradation (Chao 1), and N cycling (Shannon and Simpson index) exhibited a significant positive association with SMF (Figure 4). Conversely, the α-diversity (Simpson index) of P cycling genes showed a significant negative correlation with SMF (*p* < 0.05). In subcrust layers, only the α-diversity of P cycling genes exhibited a significant association (negative) with SMF; α-diversity indices for C- and N-related genes showed no significant relationships.

Examining correlations by biocrust types revealed consistent negative associations between C fixation gene α-diversity and SMF across all three types. C-degrading functional genes α-diversity showed consistent positive associations (Shannon and Simpson indices). For N cycling genes, significant positive correlations were observed only in moss crusts. P cycling gene α-diversity exhibited divergent correlations: positive in algal crusts but strongly negative in moss crusts.

**Figure 5:**
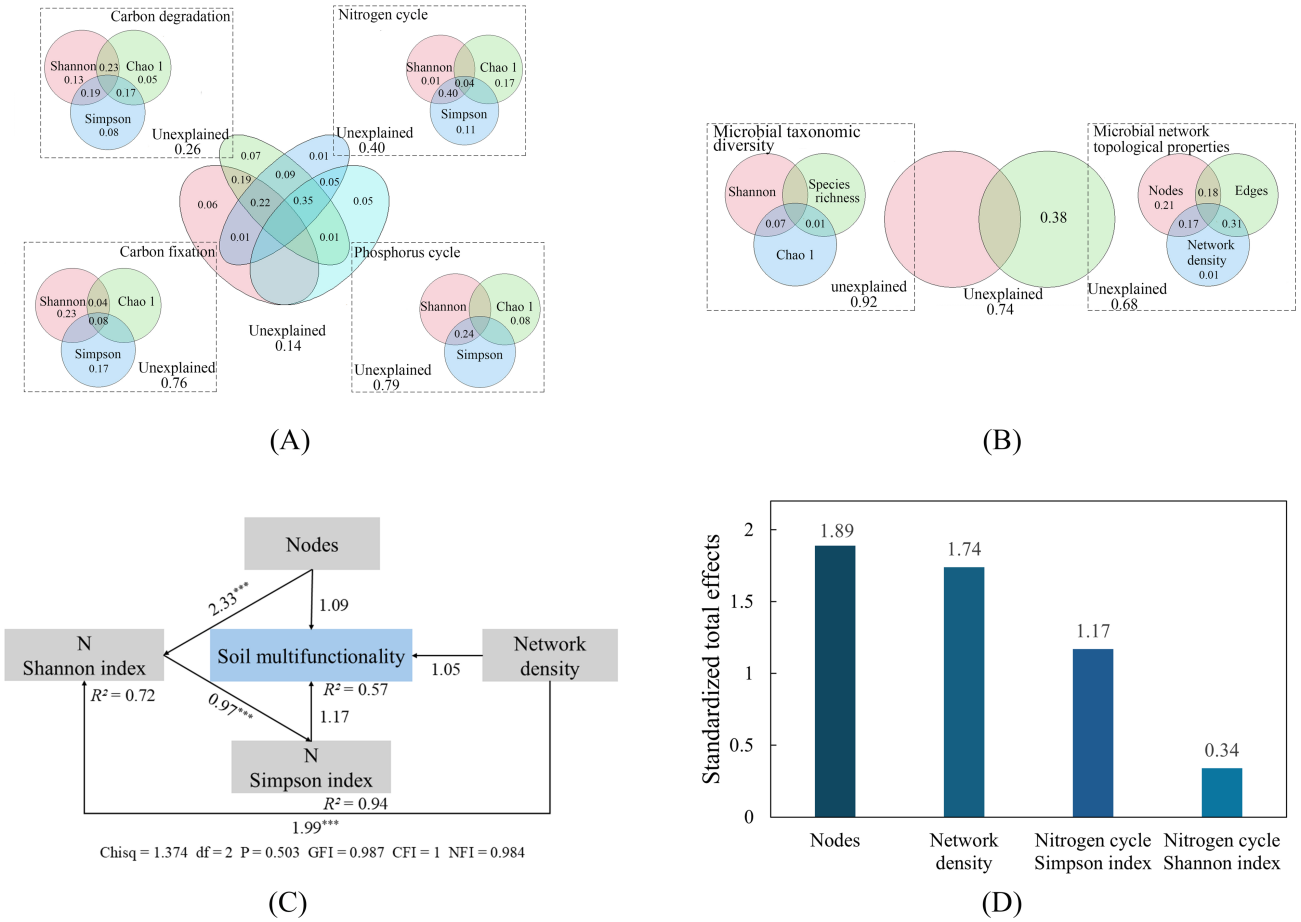
Percentage explanation of functional properties **(A)** and microbial taxonomic **(B)** on SMF (variance partition analysis); structural equation modeling **(C)** and standardized total effect **(D)** of microbial parameters on SMF. Arrows represent positive correlations, the value on the arrow is the standardized path coefficient and the degree of correlation, and *R*^2^ represents the degree of explanation of the model for the variable; GFI, Goodness fit index; CFI, Comparative fit index; NFI, Normed fit index; * *p* < 0.05; ** *p* < 0.01; *** *p* < 0.001.

Variance partitioning analysis (VPA, Figures 5A,B) revealed that functional genes diversity (C degradation, C fixation, N cycling, and P cycling) collectively accounted for 86% of the variance in SMF. Among them, C degradation gene diversity contributed the most to SMF, explaining up to 74% of the variation, followed by N cycling gene diversity at 60%. VPA also indicated that microbial taxonomic diversity and network topology together explained 26% of the variance in SMF; this explained variance was attributable solely to network topology.

Structural equation modeling (SEM, Figure 5C) explained 57% of the variance in SMF (*R*^2^ = 0.57). Key drivers identified by the SEM included microbial network node number, network density, and the α-diversity (Shannon and Simpson indices) of N cycling functional genes. The model indicated significant paths where microbial network topology positively influenced N cycling gene α-diversity, which in turn was positively associated with SMF. The SEM exhibited excellent fit: *Chisq* = 1.374 (*df* = 2, *p* = 0.503), indicating a nonsignificant discrepancy with observed data. This was corroborated by a GFI of 0.987, along with other fit indices confirming strong model performance, demonstrating robust explanatory power and reliability.

## 4 Discussion

### 4.1 Soil multifunctionality dynamics: vertical stratification and biocrust type differentiations

#### 4.1.1 Biocrusts enhance soil multifunctionality across stratified soil layers

This study demonstrated that SMF increased significantly in both biocrust layers and subcrust soils during biocrust development and succession (Figure 1). This pattern indicates a vertical facilitation effect of biocrusts. This result aligns with observations from the Gurbantunggut Desert (Huang et al., 2023). The elevated SMF in biocrust layers primarily arises from two key mechanisms: (1) atmospheric deposition and capture of dust, nutrient-rich litter, and moisture facilitated by biocrust structures (Drahorad et al., 2022); and (2) synergistic enhancements in carbon and nitrogen sequestration, phosphorus mineralization rates, enzymatic activities, soil structure, and microbial diversity and activity (Sun et al., 2020).

#### 4.1.2 Differences in soil multifunctionality among different biocrusts

Our findings demonstrate that SMF follows the order moss crusts > lichen crusts > algal crusts. The superior multifunctional performance of moss crusts stems from four reasons: (1) Carbon-driven nutrient accumulation, previous research reveals that moss crusts exhibit higher photosynthetic carbon fixation rates (51.57 g C m^−2^ yr.^−1^) compared to algal (30.64 g C m^−2^ yr.^−1^) and lichen crusts (32.71 g C m^−2^ yr.^−1^) (Feng, 2014). SOC content beneath moss crusts was 5.9-fold and 2.0-fold greater than under algal and lichen crusts, respectively (Figure 1). High carbon fixation rates significantly increase soil total carbon content while simultaneously elevating nutrient levels such as total nitrogen and available phosphorus, thereby improving the soil environment and enhancing multifunctionality (Yan et al., 2025). (2) The rhizoid network of moss plants enhances erosion resistance through microaggregate formation. These microaggregates significantly elevate soil organic carbon, total nitrogen, and total phosphorus content (Zhao et al., 2006). This process not only mitigates erosion but also enhances soil multifunctionality by modulating microbial community composition and activity (Cheng et al., 2020). (3) Moss crusts exhibited substantially higher mean soil water content (9.33%) compared to lichen (2.35%) and algal crusts (0.85%). This hydrological advantage stems from their greater specific surface area and elevated fine-particle content, which collectively enhance capillary forces (Cheng et al., 2018). The resultant improvement in water adsorption capacity promotes microbial activity and accelerates carbon-nitrogen cycling processes, thereby directly amplifying SMF (Lei et al., 2024). (4) The rapidly decomposing litter of moss crusts facilitates humus formation. This humus provides bioavailable carbon substrates that stimulate microbial metabolism, elevating soil carbon, nitrogen, and phosphorus concentrations. Consequently, accelerated nutrient cycling enhances soil multifunctionality through synergistic biogeochemical feedback (Cheng et al., 2019; Lei et al., 2024).

### 4.2 Relationship between soil microbial community characteristics and soil multifunctionality

Our results indicated that among biocrust types, only algal crusts showed a significant positive correlation between microbial taxonomic diversity and SMF, whereas no such association occurred in lichen or moss crusts. In contrast, network complexity exerted a significantly stronger influence on SMF across all biocrust types. This pattern likely arises because microbially driven processes depend less on the additive effects of microbial abundance than on complex interspecies interactions that generate integrated metabolic processes (Morrison et al., 2020). Such interactions enhance cross-functional resource utilization and metabolic coordination, thereby supporting ecosystem multifunctionality. Collectively, these findings suggest that tightly connected microbial networks underpin higher ecosystem functioning (Morrien et al., 2017).

The lack of correlation between taxonomic diversity and SMF in lichen and moss biocrusts may stem from three factors: (1) Functional antagonism among microbial taxa, where archaeal richness negatively correlates with SMF, counterbalancing positive bacterial/fungal contributions (Wang et al., 2024); (2) Aridity-driven function decoupling, as regional aridity index (0.5–0.8) weakens microbial diversity-function linkages while amplifying plant diversity dominance (Hu et al., 2021); (3) Environmental context dependency, where SMF-diversity correlations turn positive when environmental factors jointly enhance (or suppress) both properties, but become negative/non-significant under opposing environmental pressures (Rillig et al., 2019). Conversely, algal crusts maintained a positive diversity-SMF relationship, potentially attributable to their significantly higher Shannon diversity (Figure 2). This elevated richness may enable synergistic functional complementarity (Bowker et al., 2010).

Among biocrust types, lichen crusts demonstrated the highest species richness and network complexity, consistent with observations from the Gurbantunggut Desert (Zhang B. C. et al., 2018; Zhang B. et al., 2018). Three key mechanisms collectively drive this pattern: (1) Lichen acid secretion promotes soil particle cementation, while fungal hyphae bind soil particles and encapsulates microorganisms. This dual mechanism not only enhances soil aggregate stability and erosion resistance but also reduces the loss of keystone microbial taxa, thereby strengthening the resilience of microbial networks to environmental disturbances. Consequently, it ensures dual structural stability in both soil physical architecture and microbial community composition (Tumur et al., 2006; Zhang and Wang, 2010). (2) Symbiosis-mediated functional complementarity, where fungal-photobiont consortia develop complex networks via resource partitioning (hyphal protection/photosynthetic C fixation/secondary metabolite exchange) (Spribille et al., 2016). (3) Succession-dependent complexity optimization, peaking in mid-late stages through synergistic functional redundancy and keystone species coexistence, then declining due to keystone taxa loss (Wang et al., 2021; Jiao, 2023).

The biocrust layer demonstrates significantly greater network complexity than subcrust soil, confirming its critical role in augmenting soil stability. This emergent complexity is associated with the co-occurrence and potential interplay of two key factors: (1) Nutrient-enzyme co-drivers: Elevated nutrient availability coupled with enhanced enzymatic activity in the biocrust layer supplies sustained metabolic energy, facilitating coordinated microbial network assembly; (2) Heterogeneity-mediated adaptive selection: Exposure to steep spatiotemporal gradients of temperature, moisture and nutrients drives the evolution of diversified microbial survival strategies, amplifying interaction complexity through niche partitioning (Ding et al., 2023; Yang et al., 2025).

### 4.3 Relationship between soil microbial functional properties and soil multifunctionality

Research revealed a significant negative correlation between C-fixing functional gene diversity and SMF (Figure 4A). This pattern is primarily attributable to chemoautotrophic C-fixing microorganisms, which dominate subcrust microenvironments through high abundance and diversity, thereby suppressing heterotrophic microbial activity and disrupting functional equilibrium (Lynn et al., 2017). Furthermore, under environmental stress, competitively dominant C-fixing bacteria suppress other functional groups, driving increased gene diversity but decreased SMF (Chen et al., 2022). Conversely, a strong positive correlation exists between C-degrading functional gene diversity and SMF (Figure 4B). Biocrust types exhibit differential C utilization: algal and lichen crusts primarily assimilate microbially derived carbon, while moss crusts rely more heavily on autochthonous litter and microbial necromass carbon. The microbial growth-death-decomposition cycle sustains community activity via C degradation metabolism (He et al., 2024). Critically, during biocrust succession, accumulating necromass and litter drives the enrichment of C-degrading functional microorganisms, facilitating organic matter mineralization, nutrient liberation, and soil fertility enhancement (Li et al., 2024).

During biocrusts development, N cycling functional gene diversity increased significantly and exhibited a strong positive correlation with SMF (Figure 4C), primarily mediated through N-driven regulation of N transformation, organic matter turnover, and nutrient provisioning. Specifically, enriched nitrate assimilation, nitrification, and dissimilatory nitrate reduction genes synergistically enhanced N cycle efficiency (Shu et al., 2024), while elevated functional gene diversity sustained nitrogen metabolism under carbon-limited conditions via functional complementarity-driven energy allocation strategies (Zhu et al., 2023; Ma et al., 2024). Concurrently, functional redundancy conferred resilience against disturbances, when environmental stressors (e.g., pH decline) suppress specific denitrification genes, compensatory mechanisms maintained transformation efficiency (Hao et al., 2022). Furthermore, multifunctional keystone genes (e.g., *phoD*) critically amplified multifunctionality through coordinated N-P co-activation (Jia et al., 2025), collectively demonstrating how N cycling infrastructure orchestrates enhanced SMF. A context-dependent relationship was observed between the diversity P cycling functional genes and SMF across biocrust types and soil layer. Specifically, algal crusts exhibited a positive correlation, whereas moss crusts showed a negative correlation. Spatially, a negative correlation dominated in the biocrust layer, shifting to positive correlation in the subcrust layer (Figure 4D). These patterns arise from distinct mechanistic driver: (1) The higher P availability in moss crusts (compared to algal crusts) potentially suppresses the expression of some P cycling functional genes via negative feedback, inducing microbial functional trade-offs (Liu et al., 2020). Under these conditions, microbes enhance P acquisition concurrently impairing other processes, ultimately leading to a decline in multifunctionality (Delgado-Baquerizo et al., 2016). (2) Low pH in the moss crust layer reducing the abundance and diversity (Shannon index) of genes involved in inorganic P solubilization (e.g., *gcd*), further strengthening the negative correlation within this layer (Liao et al., 2023). In contrast, algal crusts, as an earlier stage of biocrust succession, exhibit lower functional gene diversity compared to moss crusts (Supplementary Figure S3). Consequently, an increase in P cycling functional gene diversity in these biocrusts tends to directly enhance nutrient supply and promote multifunctionality. Furthermore, P availability per se, rather than the diversity of functional genes, appears to be the key factor in alleviating microbial P limitation and promoting soil functions (Peng et al., 2023).

### 4.4 Microbial network complexity outweighs taxonomic diversity in driving soil multifunctionality in arid ecosystems

Our integrated analysis reveals a fundamental principle governing SMF in arid ecosystems: the synergistic interactions among microbial network topological properties and functionality supersedes taxonomic diversity as the primary driver. SEM (Figures 5C,D) further elucidated the mechanistic pathways: network topology (specifically node and network density) not only directly enhances SMF but also exerts significant indirect effects by boosting the diversity of N cycling functional genes. Critically, high network density enhances microbial synergies, significantly amplifying N-transformation efficiency. This empirically confirms that microbial network complexity underpins the sustainability of SMF (Faust and Raes, 2012).

This work provides quantitative evidence for the superior contribution of network topological properties over taxonomic diversity, identifies N cycling as a key functional module amplified by network complexity, furnishes direct mechanistic evidence linking network density to enhanced N-transformation efficiency, and underscores the pivotal role of microbial network topological properties. Given the inherent structural complexity of arid ecosystems, land management strategies should prioritize fostering conditions that build and stabilize dense, cooperative microbial functional networks, moving beyond the conventional paradigm focused solely on maximizing taxonomic diversity. Management practices should specifically target the coordinated regulation of the N cycle, recognizing this functional module as central to maintaining soil health and multifunctionality. Future research should validate the generality of the ‘network complexity-N cycling-SMF’ framework across diverse biomes and employ manipulative experiments to directly test the causal relationships between network structure, N cycling processes, and SMF.

## 5 Conclusion

This study investigated algal, lichen, and moss crusts in the Mu Us Desert to elucidate how microbial community composition and functional traits govern SMF. Moss crusts exhibited the highest level of SMF, followed by lichen and algal crusts. Compared to microbial taxonomic diversity, network topological properties exerted a stronger influence on SMF, particularly through the indirect effects mediated by network complexity (node and edge numbers). Critically, functional gene diversity metrics accounted for up to 96% of the variation in SMF, primarily driven by the diversity of genes involved in carbon degradation and nitrogen cycling. Overall, biocrusts enhanced SMF by mediating the formation of high-density microbial networks that synergistically enhanced nitrogen cycling gene diversity. These findings highlight the crucial roles of microbial network architecture and functional gene diversity in driving SMF within biocrust systems, providing important insights for managing dryland ecosystem functions and services, evaluating soil resources, and developing sustainable land-use strategies.

## Data Availability

The research data is available on Mendeley Data, V2, doi: 10.17632/cs37fxxptr.2 (Link: https://data.mendeley.com/drafts/cs37fxxptr).
