# Supplementary material for: Microbial network properties and functional gene diversity drive soil multifunctionality during biocrust succession
Source: Front Microbiol. 2025 Sep 4;16:1656706. doi: 10.3389/fmicb.2025.1656706 (PMC12443819; doi:10.3389/fmicb.2025.1656706)
Supplement: Supplementary file 1 [file Data_Sheet_1.ZIP › Supplementary Material.docx]

Supplementary Material

# **Supplementary Figures and Tables**

## **Supplementary Figures**





**Supplementary Figure 1.** Indicators of soil quality for different biocrusts. Different lowercase letters indicate significant differences (*P* < 0.05), data are shown as mean ± SE.



 **Supplementary Figure 2.** Relationship between soil multifunctionality(Z-score) and soil microbial taxonomic diversity and network topological properties. Shadow regions indicate 95% confidence intervals around the regressions. *R^2^*, Coefficient of determination; * *P* < 0.05; ** *P* < 0.01.(A): soil microbial taxonomic diversity, (B): network topological properties.





**Supplementary Figure 3.** Soil microbial functional diversity in different biocrusts. Different lowercase letters indicate significant differences (*P* < 0.05), data are shown as mean ± SE. (A): carbon fixation, (B): carbon degradation, (C): nitrogen cycle, (D): phosphorus cycle, respectively.
